# The effects of fractional CO2 laser, Nano-hydroxyapatite and MI paste on mechanical properties of bovine enamel after bleaching

**DOI:** 10.4317/jced.54044

**Published:** 2017-12-01

**Authors:** Horieh Moosavi, Narjes Hakimi

**Affiliations:** 1 Associate Professor, Dental Materials Research Center, Department of Operative Dentistry, Mashhad Dental School, Mashhad University of Medical Sciences, Mashhad, Iran; 2 Post graduate student, Dental Materials Research Center, Department of Operative Dentistry, Mashhad Dental School, Mashhad University of Medical Sciences, Mashhad, Iran

## Abstract

**Background:**

This study investigated the effect of post bleaching treatments to the change of enamel elastic modulus and microhardness after dental bleaching *in- vitro*.

**Material and Methods:**

Fifty bovine incisor slab were randomly assigned into five groups (n=10). The samples were bleached for three times; 20 minutes each time, by 40% hydrogen peroxide. Next it was applied fractional CO2 laser for two minutes, Nano- hydroxy apatite (N-HA) and MI-paste for 7 days and 2 minutes per day. The sound enamel and bleached teeth without post treatment remained as control groups. The elastic modulus and microhardness were measured at three times; 24 hours, 1 and 2 months. Data were statistically analyzed by two-way analysis of variance with 95% confidence level.

**Results:**

Different methods of enamel treatment caused a significant increase in elastic modulus compared to bleached group (*P*<0.05). Modulus was significantly increased in 1 and 2 months (*P*<0/001: bleach, *P*= 0/015: laser, *P*= 0/008: NHA, *P*=0/010: MI paste) but there were no significantly difference between 1 and 2 months (*P*>0.05). There was any significance difference for hardness among treated and control groups, but hardness increased significantly by increasing storage time (*P*<0.05).

**Conclusions:**

The use of the protective tested agents can be useful in clinical practice to reduce negative changes of enamel surface after whitening procedures.

** Key words:**Bleaching enamel, CO2 laser, MI pastes, Nano-hydroxy apatite, Microhardness, Elastic modulus.

## Introduction

Tooth whitening is one of the most common requested in dental procedures by the people (1). Tooth Bleaching is the most easy, most widespread, least invasive and most cheap than other whitening treatments (2). Mechanism of action whitening is included: the use of a chemical agent that oxidation the organic pigmentation of tooth structure (3). In addition to bleaching destroys the pigment molecules, it can also damage tooth structure (4). Evaluation of fracture toughness on the enamel surface, hardness and abrasion studies on enamel done to assessment of changes after bleaching (5). To the end of improving clinical treatment planning, it is important we have an enough science about the mechanical properties of tooth structure during bleaching (6). Micro- and nano-mechanical studies of enamel express that bleaching agents contain hydrogen peroxide (HP), significantly decreases the hardness and modulus of elasticity of enamel (4). The loss of mechanical properties of enamel after the use of bleaching agents could be regained by the incorporation of agents that can compensate for the mineral loss (7). Several types of lasers, with different parameter settings, have been used for enamel reinforcement (8). The CO2 laser (9.3, 9.6, 10.3 and 10.6 μ wavelengths), however, should be considered the mainstay in enamel hardening, because the absorption bands of phosphate, carbonate and hydroxyl groups of enamel and dentin structures are within 9.0 to 11.0 μ region which coincide well with the wavelengths of the CO2 laser (8). Recently, synthetic nano-hydroxyapatite (N-HAP) has been considered as an interesting biocompatible and bioactive material (9). It is also an important bioceramic for medical and dental applications (10). It is bioactive agent that contains calcium nanophosphate organized in a crystalline form of hydroxyapatite (11). N-HAP has some potential to repair enamel (12). A new technology based on casein phosphopeptide- amorphous calcium phosphate (CPP-ACP) has been recently introduced. The aim of this technique is to create a calcium and phosphate reservoir that can bind stably to plaque and dental surfaces (13). It has been reported that the application of a CPP-ACP paste (MI paste) either before and after in-office bleaching protocols was able to prevent negative changes of roughness and hardness to bovine enamel (13). The aim of this study was to investigate the effects of fractional CO2 laser irradiation, Nano-hydroxyapatite and MI paste on mechanical properties of enamel after bleaching.

## Material and Methods

-Specimen preparation

Freshly extracted bovine incisor teeth without visible caries or structural defects on enamel surface were collected, cleaned, and disinfected in a 0.1 % thymol solution at room temperature. Enamel specimens (4×4×2 mm3) were prepared from the labial aspects and embedded in epoxy resin. The labial surfaces were wet-polished using aluminum oxide abrasive papers (600-800 -1000 -1500 grit and 2000- grit) to produce flat enamel. Samples stored in inartificial saliva during the whole experimentation.

**Figure 1:**
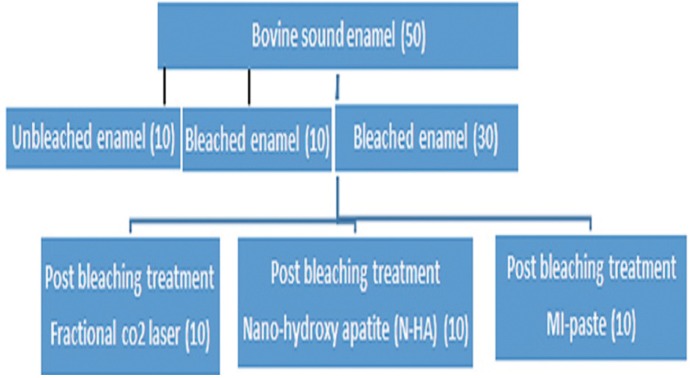
Experimental samples flowchart.

-Surface treatment:

The teeth were randomly allocated into five groups (n=10). Sound enamel (SE): samples were left untreated and served as the negative control group. Bleaching (B): application of 40% HP (Opalescence Ultradent Products, Inc, South Jordan, UT, USA) three times, each time 20 minutes as the positive control group. Bleaching and Laser (BL): Enamel was first treated with HP and then exposed to irradiation from a fractional CO2 laser (wavelength 10.6 μm; Lutronic Inc., Princeton Junction, NJ, USA). The laser was operated in the dynamic mode with frequency of 200 Hz, 10 mJ of energy, and power of 10 W, and the beam was adjusted to cover a square area of 4×4 mm2 for 10 s per tooth. The laser’s handpiece was held manually at an approximate distance of 25 mm from the sample surface by one investigator. Bleaching and N-HA (BNHA): samples in the group were subjected to HP and then applied Nano-HA cream for 7 days and 2 minutes per day with a brush. Bleaching and MI paste (BMI): specimens were whitened with HP then exposed to MI paste (GC America) for 7 days and 2 minutes per time with a carrier. The schematic view of experimental samples flowchart was presented in figure 1. The condition for storage between tests and treatments was artificial saliva and incubation at 37 °C and humidity of 100%. Twenty four hours, 1 month and 2 months after last treatment, the specimens were tested by elastic modulus and hardness.

-Elastic modulus Assessment

The samples were put on the table of the universal testing machine (BONGSHIN korea). Following this, a compressive force was applied with a round end stainless steel of 0.13 mm diameter at the end, crosshead speed of 0.5 mm/minute.

-Microhardness Assessment

A micro Vickers hardness tester (Koopa MH3, Iran) was utilized under a load of 100 gr and a well time 10 seconds for microhardenss measurement, placing its indenter at the labial enamel surface, and repeated at three points.

-Scanning electron microscopic examination 

The specimens dehydrated with 50%, 70% and 85% alcohol, then coated with gold-palladium alloy and then analyzed under scanning electron microscopy (SEM: vp 1450 leo, Germany). Each specimen at 5000x original magnification was obtained.

-Statistical analysis:

Mean ± SD was used as descriptive statistics of elastic modulus and hardness. Because of interaction between time and groups repeated measure ANOVA was done for comparison of elastic modulus along the time in each group and one-way ANOVA was done to compare elastic modulus among groups in each time. Repeated measure ANOVA was used to evaluate effects of time and group on hardness. Pairwise comparison was carried out to calculate the odds ratio. The significance level was set at 0.05.

## Results

**Figure 2:**
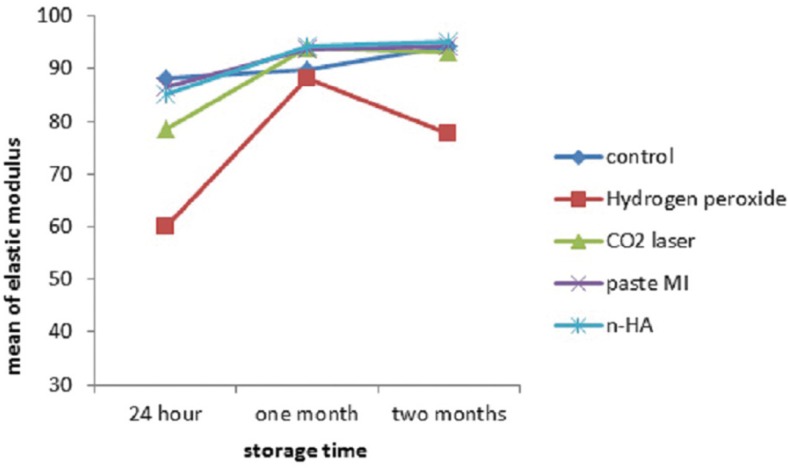
Mean of elastic modulus in each group after 24 h, 1 month and 2 months.

The maximum and minimum elastic modulus was seen in the BNHA after 2 months and bleached after 24 hours groups respectively ( Table 1, Fig. 2). Repeated measure ANOVA showed that only in control group mean of elastic modulus didn’t change statistically significant along the time (*P*=.42) and in all other groups change of elastic modulus was statistically significant along the time (*P*<.05). Post-Hoc test of LSD with Bonferroni correction showed that in hydrogen peroxide, laser CO2, MI paste and n-HA groups, elastic modulus was statistically different between 24 h and 1 month and 24 h and 2 months (*P*<.05). One-way ANOVA showed that 24 h and two months after the beginning of the study elastic modulus were statistically different among groups (*P*<.001). Post-hoc test of Tukey showed that 24 h and 2 months after the beginning of the study elastic modulus of hydrogen peroxide were statistically different with all other groups (*P*<.05).

**Table 1:**
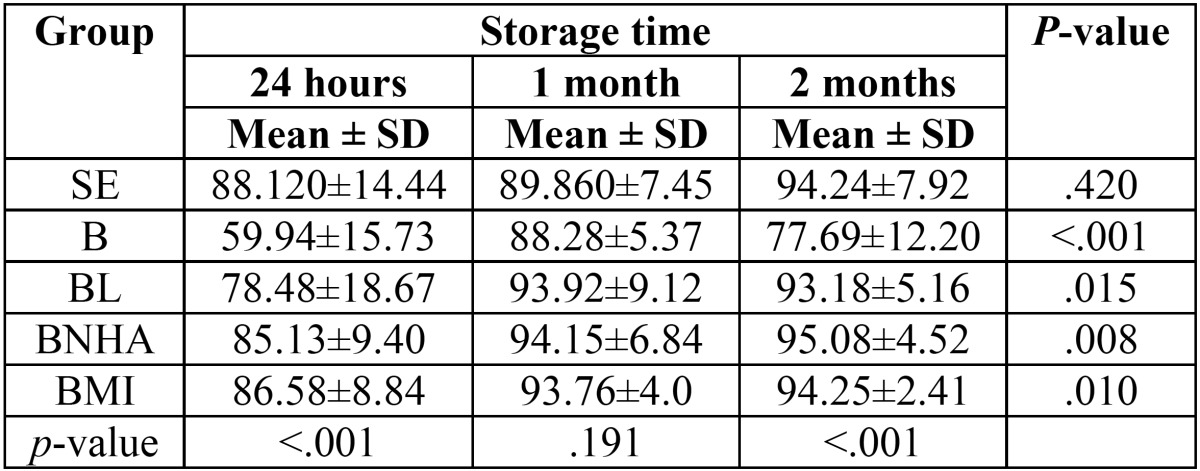
Mean ± SD of elastic modulus in each group after 24 hours, 1 month and 2 months.

**Figure 3:**
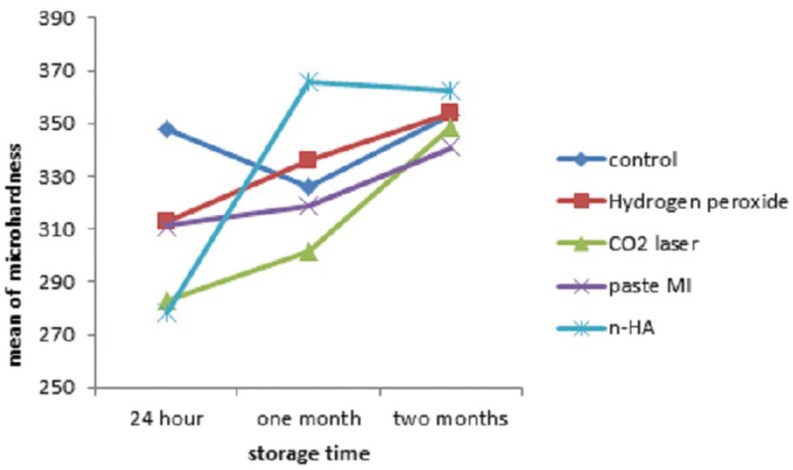
Mean of hardness in each group after 24 hours, 1 month and 2 months.

Repeated measure ANOVA showed that mean of hardness was not statistically different among groups (F=1.245, P=.295) but that variable changed statistically significant along the time (F=7.177, *P*<.001). Post-Hoc test of LSD with Bonferroni correction showed that mean of hardness was statistically different between 24 h and 2 months (*P*<.05) ( Table 2, Fig. 3).

**Table 2:**
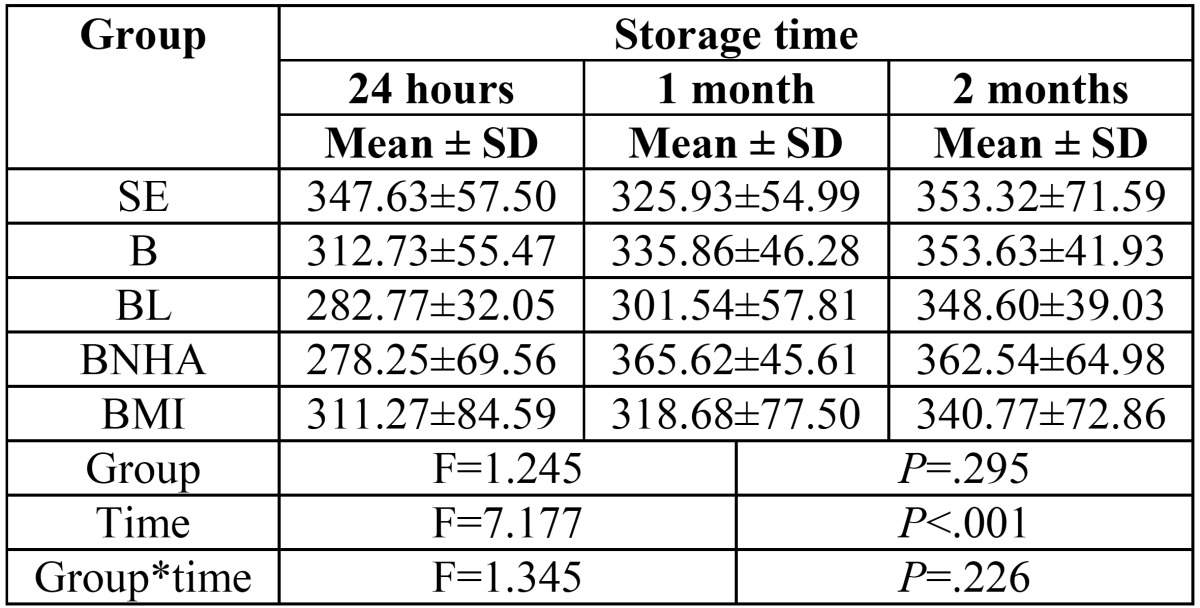
Mean ± SD of hardness in each group after 24 h, 1 month and 2 months.

**Figure 4:**
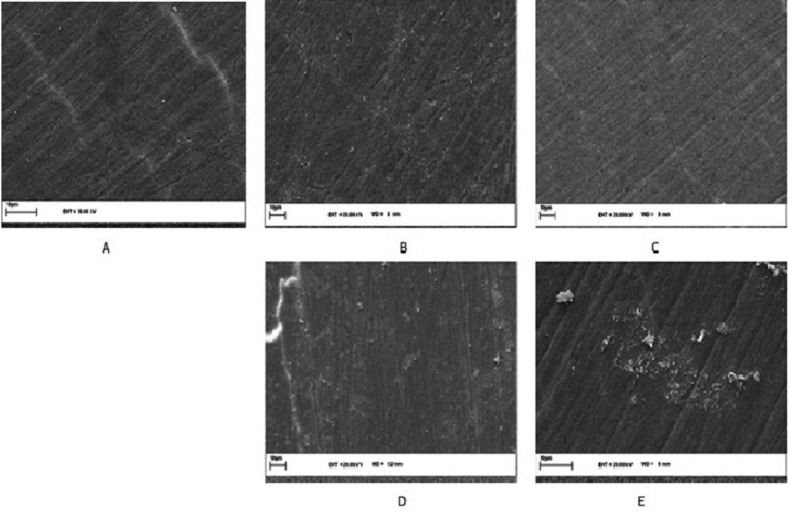
A) Photomicrograph of SEM (5000x) sound enamel, B) SEM image (5000x) of B group after 24 hours. The bleaching agent caused porosities, depressions, and superficial alterations at various degrees. C) BL group, D) BNHA group, E) BMI group.

**Figure 5:**
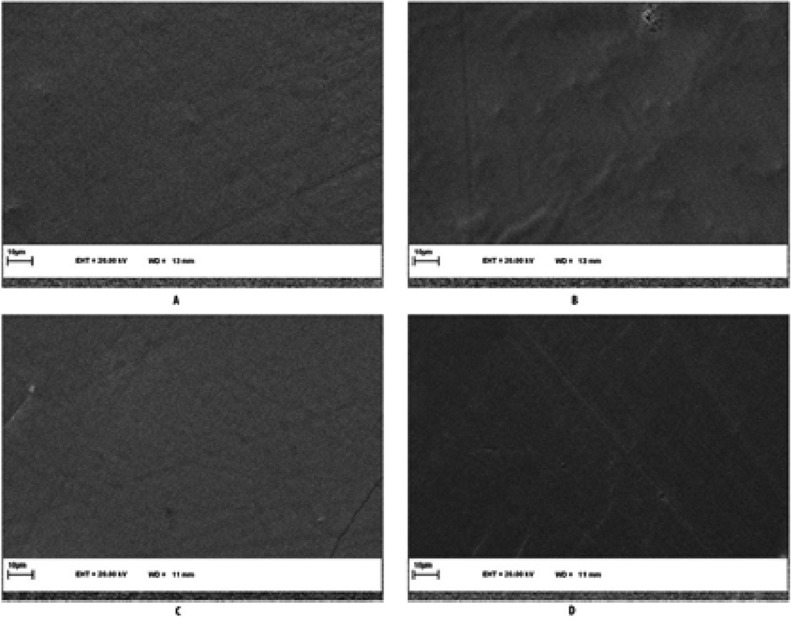
SEM image (5000x) treated groups after 2 months. A) B group, B) BL group, C) BNHA group, D) BMI group after 2 months.

Also SEM revealed difference in the morphology depends on the time and treatment protocol figure 4 and 5.

## Discussion

Reduced enamel surface mechanical properties and increased hypersensitivity as a result of bleaching treatments are matters of concern. Enamel hardness and hypersensitivity are associated with the removal of mineral content from the enamel and dentine (14). In order to avoid the possible adverse effect and to overcome mineral loss due to bleaching procedures, different method and materials of restructuring bleached dental enamel have been suggested. The measurement of surface hardness is frequently used to evaluate the effects of bleaching agents on dental hard tissue (14). In the present study, a bleaching agent containing a high concentration (40%) of HP (PH 3.7) was used to obtain the maximum negative effect on the enamel and followed by evaluation of microhardness and elastic modulus after 24 hours, 1 and 2 months. In the current study, it was used three different times to evaluation of modulus, hardness and surface morphology and it showed that there are significant differences through the passage of time. Oshiro used the fourth period to evaluate the elastic modulus, that results revealed rising the modulus during the one month of experimental period (6). While other studies have used less time courses (4,8,15). Fractional CO2 laser, N-HA and MI paste and was used in this study for treatment of bleached enamel. It seems that fractional CO2 laser following absorption, there would be a high temperature increase in the surface and near the surface enamel layers, which results in structural and chemical alterations of enamel including decomposition of organic matrix, reduction of carbonate content, and fusion and recrystallization of hydroxyapatite crystals (8,15,16) N-HA and MI paste enhance the remineralization of enamel surface, which had been demineralized after tooth bleaching (8,17). Nano-hydroxyapatite enhances remineralization of enamel surfaces by occluding the surface microporosities, which results in prevention stain absorption and increasing surface microhardness (18). Very few studies have been done on the effect of post bleaching treatment on elastic modulus of enamel. In this study the elastic modulus of enamel after bleaching reduced and after treatment it was more than bleached enamel. Other studies have shown that 35% HP reduced elastic modulus of enamel and it’s because of destruction of protein matrix by the peroxide free radicals (4) and treatment after bleaching increase modulus (6) and protects the negative change of enamel structure (19). It seems that fractional CO2 laser, N-HA and MI paste was effective in increase the elastic modulus of enamel. The laser treatment was performed only once in the present *in vitro* study and a high elastic modulus were achieved. This could be an advantage for the treatment of patients suffering from sensitivity after bleaching, because it would not involve dependence on frequent use of demineralization paste (20). It was concluded that increase in elastic modulus of bleached enamel could be achieved by post treatment. Bleaching procedures also involve different techniques with different composition of peroxides (21). Literature quotes numerous studies showing adverse effects of these agents on tooth microhardness (4,8). In our study the specimen were exposed to 40 % HP. Our findings were in accordance with the results obtained by previous studies who noticed no enamel alteration after bleaching solution exposure (21,22). In this study there was significant difference between 24 hours and two months and hardness was higher in two months. According to a study, there was any difference between 24, 48 hours and 1 week of bleached groups (21). Probably the use of remineralization agents leads to this difference. The use of pulsed (from 50 to 100 ps) CO2 laser irradiation causes surface melting and crystal fusion at fluencies well below those quoted above for CO2 irradiation, without accompanying ablation or undesirable subsurface heating (15,23). The nano-crystals of phosphate are smaller than 100 nm, improving the bioactivity of agent due to the increase in the superficial area and wettability of hydroxyapatite nanoparticles. The calcium, phosphate, and fluoride ions released might increase the saturation level of liquid adjacent to the dental surface , thus promoting remineralization (11). It has been reported that the application of a CPP-ACP paste either before or before and after in-office bleaching protocols was able to prevent negative changes of roughness and hardness to bovine enamel (17). Under the conditions of this in-vitro study, 40% hydrogen peroxide bleaching gel application decreased the elastic modulus and hardness of bovine enamel. However, bleaching followed by MI paste, fractional CO2 laser and N-HA application led to an increase in the elastic modulus and hardness of bleached enamel. However, the reduction in mechanical properties following bleaching treatment can be controlled by the action of saliva in clinic or artificial demineralizing agents. In the current study, the enamel surfaces by different treatments were examined by a scanning electron microscope. As shown in the figure 4, bleached enamel shown initial enamel lesion that formed on the surface and had significantly more porosity than the sound enamel. So the effect of hydrogen peroxide on enamel surface structure resulted in an erosion-like roughened surface. This is similar to previous studies (4,17). After treatment of bleached enamel has been shown decreased in porosity and roughness after 24 hours. Smoothness after two months was more than 24 hours. In the present study, photomicrographs showed that CO2 laser irradiation fused the surface, creating a smooth recrystallized aspect. Also, fusion between hexagonal shaped crystals of enamel surface after 10.6 l m CO2 laser treatment was also observed under SEM by Souza -Gabriel *et al.* (15). Comparing the control samples (Figs. 4,5) with bleached enamel treated with the different pastes demonstrated that utilization of all the protective agents advanced the creation of a shallow mineral layer. In these specimens some surface areas were covered by smear layer. Indeed, in spite of the fact that the blanching convention brought about a critical increment of surface abnormalities even if a protective paste was subsequently applied, bleached enamel group showed higher surface alterations, significantly different from other groups. These observations are in agreement with previous studies on the protective efficacy of CPP-ACP and Nano- hydroxy apatite pastes (17). It was proposed to do other research about the effect of various bleaching agents and remineralization factors in different interval after bleaching on mechanical properties of dentin.

## Conclusions

The utilization of the protective tested agents can be helpful in clinical practice to lessen negative changes of enamel surface after whitening strategies. Significant reduction of modulus elasticity and hardness occurred after whitening treatment that eliminated after two months.

## References

[1] Carey CM (2014). Tooth whitening: what we now know. J Evid Based Dent Pract.

[2] Bollineni S, Janga RK, Venugopal L, Reddy IR, Babu PR, Kumar SS (2014). Role of fluoridated carbamide peroxide whitening gel in the remineralization of demineralized enamel: An in vitro study. J Int Soc Prev Community Dent.

[3] Gladwell J, Simmons D, Wright JT (2006). Remineralization potential of a fluoridated carbamide peroxide whitening gel. J Esthet Rest Dent.

[4] Elfallah HM, Bertassoni LE, Charadram N, Rathsam C, Swain MV (2015). Effect of tooth bleaching agents on protein content and mechanical properties of dental enamel. Acta Biomater.

[5] Tam LE, Lim M, Khanna S (2005). Effect of direct peroxide bleach application to bovine dentin on flexural strength and modulus in vitro. J Dent.

[6] Oshiro M, Kurokawa H, Ando S, Irokawa A, Miyazaki M, Platt JA (2007). The effect of bleaching on the elastic modulus of bovine enamel. Dent Mater J.

[7] George L, Baby A, Dhanapal TP, Charlie K, Joseph A, Varghese AA (2015). Evaluation and comparison of the microhardness of enamel after bleaching with fluoride free and fluoride containing carbamide peroxide bleaching agents and post bleaching anticay application: An in vitro study. Contem Clin Dent.

[8] Moosavi H, Darvishzadeh F (2016). The Influence of Post Bleaching Treatments in Stain Absorption and Microhardness. Open Dent J.

[9] Santos LF, Torres CR, Caneppele TM, Magalhaes AC, Borges AB (2016). Effect of home-bleaching gels modified by calcium and/or fluoride and the application of nano-hydroxyapatite paste on in vitro enamel erosion susceptibility. Acta Odonto Scand.

[10] Haghgoo R, Rezvani MB, Salehi Zeinabadi M (2014). Comparison of nano-hydroxyapatite and sodium fluoride mouthrinse for remineralization of incipient carious lesions. J Dent (Tehran, Iran).

[11] de Carvalho FG, Vieira BR, Santos RLd, Carlo HL, Lopes PQ, de Lima BASG (2014). In vitro effects of nano-hydroxyapatite paste on initial enamel carious lesions. Pedia Dent.

[12] Tschoppe P, Zandim DL, Martus P, Kielbassa AM (2011). Enamel and dentine remineralization by nano-hydroxyapatite toothpastes. J Dent.

[13] Poggio C, Grasso N, Ceci M, Beltrami R, Colombo M, Chiesa M (2016). Ultrastructural evaluation of enamel surface morphology after tooth bleaching followed by the application of protective pastes. Scanning.

[14] Yesilyurt C, Sezer U, Ayar MK, Alp CK, Tasdemir T (2013). The effect of a new calcium-based agent, Pro-Argin, on the microhardness of bleached enamel surface. Aust Dent J.

[15] Souza-Gabriel A, Colucci V, Turssi C, Serra M, Corona S (2010). Microhardness and SEM after CO2 laser irradiation or fluoride treatment in human and bovine enamel. Micr Res Tech.

[16] Poosti M, Ahrari F, Moosavi H, Najjaran H (2014). The effect of fractional CO2 laser irradiation on remineralization of enamel white spot lesions. Lasers Med Sci.

[17] Cunha AG, De Vasconcelos AA, Borges BC, Vitoriano Jde O, Alves-Junior C, Machado CT (2012). Efficacy of in-office bleaching techniques combined with the application of a casein phosphopeptide-amorphous calcium phosphate paste at different moments and its influence on enamel surface properties. Microsc Res Tech.

[18] Zanolla J, Marques A, da Costa DC, de Souza AS, Coutinho M (2017). Influence of tooth bleaching on dental enamel microhardness: a systematic review and meta-analysis. Aust Dent J.

[19] Son JH, An JH, Kim BK, Hwang IN, Park YJ, Song HJ (2012). Effect of laser irradiation on crystalline structure of enamel surface during whitening treatment with hydrogen peroxide. J Dent.

[20] Esteves-Oliveira M, Pasaporti C, Heussen N, Eduardo CP, Lampert F, Apel C (2011). Rehardening of acid-softened enamel and prevention of enamel softening through CO2 laser irradiation. J Dent.

[21] Dey S, Pandey V, Kumar A, Awasthi N, Sahu A, Pujari SC (2016). In vitro Comparison of Impact of Different Bleaching Agents on the Microhardness of Enamel. J Contemp Dent Pract.

[22] Smidt A, Weller D, Roman I, Gedalia I (1998). Effect of bleaching agents on microhardness and surface morphology of tooth enamel. Am J Dent.

[23] McCormack S, Fried D, Featherstone J, Glena R, Seka W (1995). Scanning electron microscope observations of CO2 laser effects on dental enamel. J Dent Res.

